# Recombination between Polioviruses and Co-Circulating Coxsackie A Viruses: Role in the Emergence of Pathogenic Vaccine-Derived Polioviruses

**DOI:** 10.1371/journal.ppat.1000412

**Published:** 2009-05-01

**Authors:** Sophie Jegouic, Marie-Line Joffret, Claire Blanchard, Franck B. Riquet, Céline Perret, Isabelle Pelletier, Florence Colbere-Garapin, Mala Rakoto-Andrianarivelo, Francis Delpeyroux

**Affiliations:** 1 Institut Pasteur, Unité de Biologie des Virus Entériques, Paris, France; 2 Institut Pasteur de Madagascar, Unité de Virologie Médicale, Antananarivo, Madagascar; Centro de Biología Molecular Severo Ochoa (CSIC-UAM), Spain

## Abstract

Ten outbreaks of poliomyelitis caused by pathogenic circulating vaccine-derived polioviruses (cVDPVs) have recently been reported in different regions of the world. Two of these outbreaks occurred in Madagascar. Most cVDPVs were recombinants of mutated poliovaccine strains and other unidentified enteroviruses of species C. We previously reported that a type 2 cVDPV isolated during an outbreak in Madagascar was co-circulating with coxsackieviruses A17 (CA17) and that sequences in the 3′ half of the cVDPV and CA17 genomes were related. The goal of this study was to investigate whether these CA17 isolates can act as recombination partners of poliovirus and subsequently to evaluate the major effects of recombination events on the phenotype of the recombinants. We first cloned the infectious cDNA of a Madagascar CA17 isolate. We then generated recombinant constructs combining the genetic material of this CA17 isolate with that of the type 2 vaccine strain and that of the type 2 cVDPV. Our results showed that poliovirus/CA17 recombinants are viable. The recombinant in which the 3′ half of the vaccine strain genome had been replaced by that of the CA17 genome yielded larger plaques and was less temperature sensitive than its parental strains. The virus in which the 3′ portion of the cVDPV genome was replaced by the 3′ half of the CA17 genome was almost as neurovirulent as the cVDPV in transgenic mice expressing the poliovirus cellular receptor gene. The co-circulation in children and genetic recombination of viruses, differing in their pathogenicity for humans and in certain other biological properties such as receptor usage, can lead to the generation of pathogenic recombinants, thus constituting an interesting model of viral evolution and emergence.

## Author Summary

Following intense vaccination campaigns with Sabin's trivalent live-attenuated oral poliovirus vaccine, poliomyelitis caused by wild polioviruses has disappeared from large parts of the world. However, poliomyelitis outbreaks due to pathogenic circulating vaccine-derived polioviruses (cVDPVs) have recently been reported in countries with low vaccine coverage. Most of these cVDPVs seem to be recombinants of mutated vaccine strains and undetermined coxsackieviruses. We have previously shown a cVDPV isolated during an outbreak in Madagascar to be co-circulating with coxsackievirus A17 (CA17) strains with 3′ genomic sequences related to those of the cVDPV. In this study, we determined whether these CA17 isolates can act as recombination partners of poliovirus. Using genetic engineering techniques, we constructed a variety of recombinant viruses derived from a CA17 isolate, the cVDPV and the corresponding original vaccine strain. Our results showed that poliovirus/CA17 recombinants are viable. Moreover, the recombinant virus resulting from the replacement of the 3′ half of the cVDPV genome by that of the CA17 genome was almost as pathogenic as the cVDPV. This supports the notion that co-circulation and co-evolution through the recombination of polioviruses and coxsackieviruses contribute to the emergence of epidemic cVDPVs. This constitutes an interesting model of viral evolution and emergence.

## Introduction

The Sabin's trivalent live-attenuated oral poliovirus vaccine (OPV) includes viral strains of three serotypes (Sabin 1, 2 and 3). These strains replicate in the digest tract following vaccination, inducing a strong local intestinal immune response that limits subsequent poliovirus (PV) replication and viral transmission in humans [1]. OPV is the main tool used in the Poliomyelitis Eradication Initiative, which was launched by the World Health Organization (WHO) in 1988 to eradicate this acute paralytic disease (http://www.polioeradication.org/). Following intense vaccination campaigns, wild polioviruses have disappeared from major parts of the world, remaining endemic in only four countries, India, Pakistan, Afghanistan and Nigeria [2]. Nevertheless, low vaccine coverage over recent years has allowed occasional spread of wild PV strains from endemic countries to neighboring or distant countries where wild PV had disappeared [3]. Moreover, low vaccine coverage can lead to transmission of OPV strains to non-immunized people, allowing genetic drift and subsequent loss of their attenuation characteristics [4],[5]. Ten outbreaks due to pathogenic circulating vaccine-derived polioviruses (cVDPVs) have recently been reported, two of which occurred in Madagascar in 2001–2002 and 2005 [3], [6]–[13].

All but one of the cVDPV outbreaks were due to recombinant lineages between vaccine PVs and other unidentified human enteroviruses of species C (HEV-C) [3], [6]–[13]. HEV-C (*Picornaviridae* family) include PVs and related coxsackie A virus serotypes [14]–[16] (http://www.ictvonline.org/). Enteroviruses are non-enveloped viruses with a single positive-strand RNA genome approximately 7.5 kb long. The single large coding region of the genome is flanked by 5′ and 3′-untranslated regions (5′- and 3′UTR). The coding region is translated as a single polyprotein, which is then processed by viral proteases to yield mature viral proteins including the capsid proteins (VP1 to VP4) and non-structural proteins [17]. In recombinant cVDPV genomes, large parts of the region encoding non-structural proteins, the 3′UTR and in some cases the 5′UTR are derived from non-poliovaccine HEV-C [5]–[7],[9],[10].

Recombination in enteroviruses was discovered in 1962 [18],[19]. The ability of enteroviruses to undergo extensive recombination is now well established, and represents another mechanism, together with mutations, by which these viruses generate diversity and evolve [20]–[29]. Recombination is also thought to be involved in repair of deleterious mutations in genomes [4],[30]. However, the role of recombination in the generation and function of recombinant strains — in particular of the newly discovered recombinant cVDPVs — remains unclear [5],[21],[22].

During the polio-outbreak in 2001–2002 in Madagascar, two different type 2 recombinant cVDPV lineages, with sequences derived from the 5′ half of the Sabin 2 genome and the 3′ half of non-PV HEV-C, were isolated. [11],[27]. Recombination sites were located in the region encoding viral protease 2A. Moreover, many coxsackie A serotypes showing substantial genetic diversity were found in the stools of children living in the small area where most of the poliomyelitis cases occurred [27]. Partial genomic sequencing showed that the sequences of several serotype 17 and 13 coxsackie A viruses (CA17 and CA13) encoding viral proteins 2C and 3D^pol^ (polymerase), respectively, were closely related to those of the cVDPV sequences. These results suggest that ancestors of these CA17 and CA13 strains were the donors of the HEV-C sequences present in the Madagascar cVDPVs; however, the nucleotide (nt) sequences showing the highest similarity between these coxsackie A viruses and cVDPVs remained significantly different (6–8%). Although these cVDPVs and coxsackie A viruses appear to have common ancestors, the observed differences in nt sequence could have been generated either before or after the recombination event. Finally, other coxsackie A viral strains or serotypes with sequences more closely-related to those of the cVDPVs may have acted as parental donors.

The infectious cDNA of one Madagascar cVDPV (MAD04) belonging to the major lineage isolated in 2002 was cloned and used with that of the original Sabin 2 vaccine strain to construct cDNA-derived recombinants [31]. The results demonstrated that the HEV-C sequences affected the characteristics of this cVDPV. In particular, although the key neurovirulence determinants of MAD04 are located in the 5′ half of the genome (mutated Sabin 2 sequences), the HEV-C-derived 3′ half of the genome affected pathogenicity. However, it is not known whether this effect was due to the primary recombination event involved in the generation of the cVDPV or to subsequent mutations optimizing the functional interactions between both halves of the genome.

We determined whether CA17 strains can be recombination partners of PV and evaluated the key effects of such a recombination event on the phenotype of the recombinants. We cloned the infectious cDNA of a CA17 isolate and used it in conjunction with that of Sabin 2 and MAD04 to construct and characterize chimeric viruses. We found that non-polio HEV-C sequences present in MAD04 can be replaced by CA17 sequences, and that CA17 sequences contribute to the phenotypic characteristics of PV/CA17 recombinants, including pathogenicity.

## Results

### Genomic sequencing and characterization of the Madagascar CA17.67591 strain

To confirm the relationship between CA17 isolates and the cVDPV MAD04 lineage, we sequenced the whole genome of one of the CA17 isolates (CA17.67591) that was found co-circulating with MAD04 [27] – EMBL Genbank accession number FM955278. The genome of this isolate is 7457 nt long, excluding the poly(A) tail. The CA17.67591 genome encodes a single open-reading frame (encoding a 2213 amino-acid polyprotein) flanked by a 747 nt 5′UTR and a 71 nt 3′UTR.

**Figure 1:**
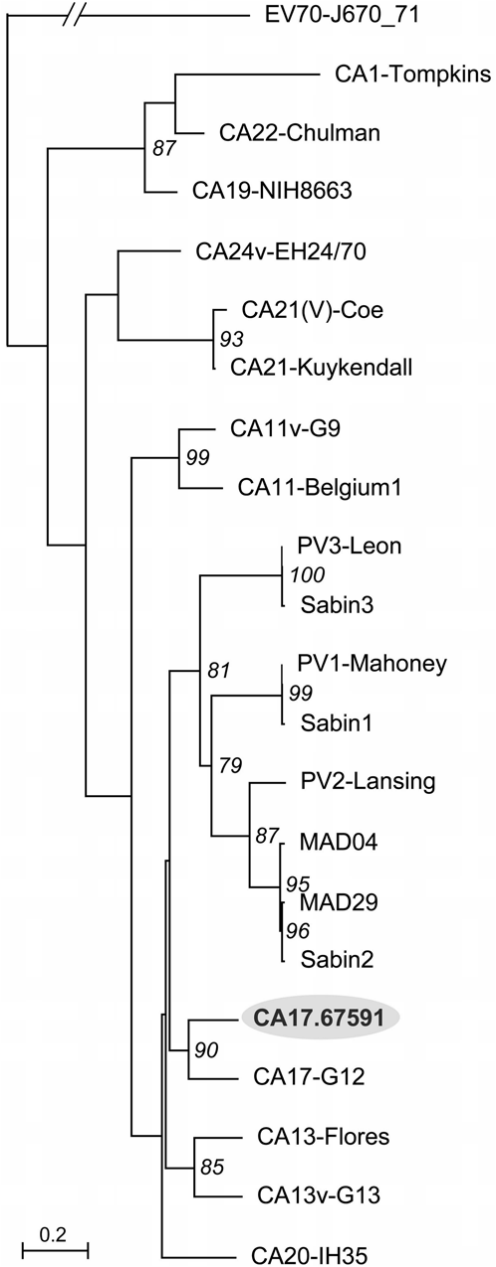
Phylogenetic tree depicting genetic relationships between nucleotide sequences of CA17.67591, cVDPVs and other HEV-C prototypes. This neighbor-joining tree was based on nucleotide sequences alignment of genomic regions P1, which encode viral capsid proteins. Branch lengths were calculated using PUZZLE and the Hasegawa, Kishino and Yano (HKY) model of substitution. The genetic distance is indicated (bar). Numbers at nodes correspond to the percentage of 10,000 puzzle steps supporting the distal cluster. Nucleotide sequences of enterovirus 70 (EV70) were used as an outgroup.

The nt sequences encoding the VP1 capsid protein of enterovirus isolates are serotype-specific [32]. In an alignment of VP1 nt sequences of CA17.67591 with those of HEV-C prototype strains, the CA17-G12 prototype strain showed the highest nt identity (78%). Alignment of the CA17.67591 VP1 polypeptide also showed high sequence similarity with the VP1 polypeptide of this prototype strain (94%). VP1-encoding genomic regions of both CA17 strains were 918 nt long and differed from those of the other prototype HEV-C strains. Given that enteroviruses of the same serotype generally have >75% nt sequence identity and >85% amino-acid similarity in VP1 regions, these results strongly suggest that the CA17.67591 strain belongs to the same serotype as the prototype strain CA17-G12 [32]–[34]. These results were confirmed by aligning and comparing P1 genomic region nucleotide sequences encoding the capsid proteins of CA17.67591 and of various HEV-C prototypes in phylogenetic trees (maximum likelihood method) (Fig. 1). P1 sequences from CA17.67591 clustered with those of CA17-G12 with high reliability values (percent puzzle steps ≥90%). Similar results were obtained using VP1 nucleotide sequences (not shown). The stem-loop structure containing the AAACA conserved motif, constituting the *cis*-acting replication element *cre(2C)*, was found in CA17.67591 at nt positions 4461–4521 [35]–[37].

Several non-PV HEV-C (CA13, CA15, CA20 and CA21) bind intercellular adhesion molecule-1 (ICAM-1), which thus acts as a viral cellular receptor (or at least a co-receptor); in most cases, the binding of these strains to cells can be blocked by anti-ICAM-1 antibodies [15],[38],[39]. The CA17 receptor is currently unknown. We therefore tested whether CA17 infection was mediated by ICAM-1, using the prototype CA17-G12 and the isolate CA17.67591. Infection of HEp-2c cells by these two CA17 strains was completely inhibited by the anti-ICAM-1 mAb 8.4 A6 [40], but not by the anti-CD155 mAb 404 [41] directed against the PV receptor (data not shown). Conversely, HEp-2c cells were completely protected from Sabin 2 infection by the anti-CD155 mAb, but not by the anti-ICAM-1 mAb. These results suggested that infection by CA17-G12 or CA17-67591 is mediated by ICAM-1, as observed for the other non-PV HEV-C.

### Comparative analysis of CA17.67591 and cVDPV sequences from Madagascar

**Figure 2:**
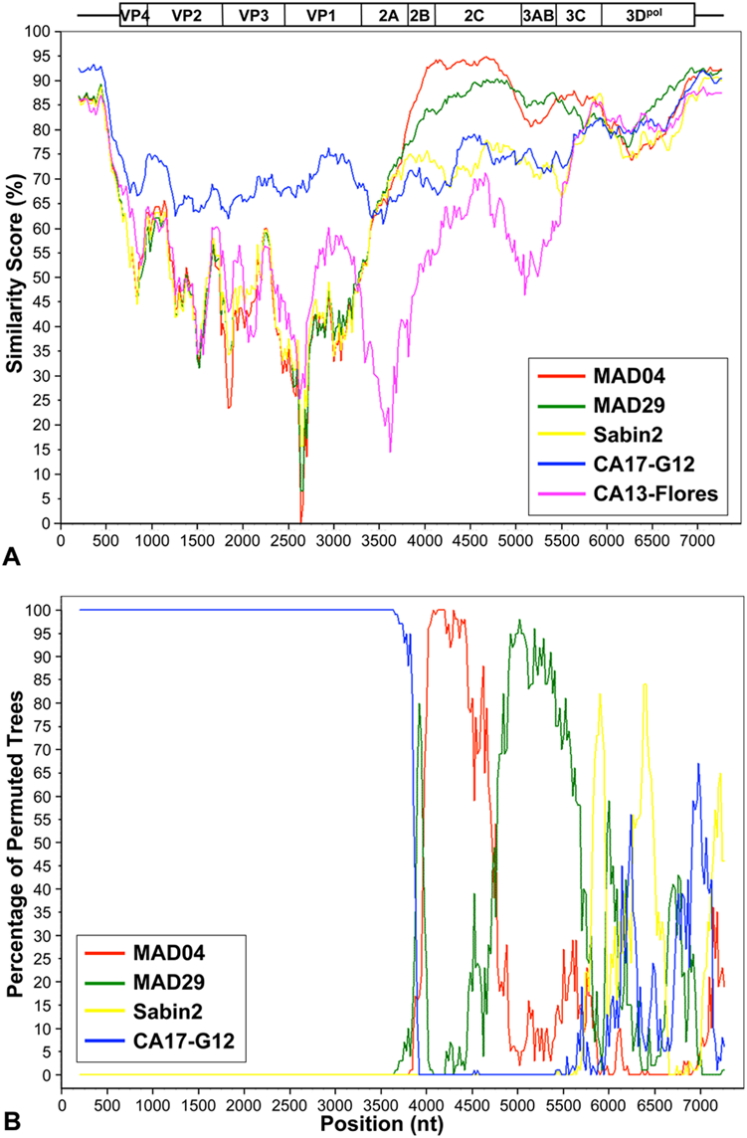
Comparative analysis of the genomic sequences of CA17.67591, cVDPVs and HEV-C prototype strains. A) Similarity between the genomic sequences of CA17.67591 and other strains: prototype strains CA17-G12 and CA13-Flores, Madagascar cVDPVs MAD04 and MAD29, vaccine strain Sabin 2. The genetic organization of the PV genome is shown. B) Bootscanning plot of MAD04, MAD29, Sabin 2 and CA17 sequences versus CA17.67591 sequences (query sequence). Similarity and bootscanning analysis were performed with a sliding window of 400 nt, with a step of 20 nt (Simplot software version 3.5.1).

We carried out an overall comparative analysis of the genomic nt sequences of the two Madagascar cVDPV lineages (MAD04 and MAD29), Sabin 2 and the prototype strains CA17-G12 and CA13 Flores, by alignment with CA17.67591 nt sequences. Pairwise comparisons were performed using similarity scanning analysis [42] (Fig. 2A). The highest similarity score was obtained for comparison of the 5′ half of the CA17-G12 sequence (5′UTR and capsid region) with the homologous regions of CA17.67591, confirming that these strains belong to the same HEV-C serotype. High similarity scores were also observed when comparing the MAD04 and MAD29 cVDPV nt sequences encoding viral proteins 2B and 2C with those of CA17.67591, This was confirmed by comparing in a more detailed analysis nucleotide sequences — and to a lesser extent, amino-acid sequences — of genomic regions of CA17.67591, with those of other HEV-C (Table 1). Similar results were previously obtained from partial sequencing of CA17.67591 [27], suggesting that ancestors of CA17.67591 and other related CA17 isolates from Madagascar were the donor strains of some of the non-poliovirus sequences present in MAD04 and MAD29.

**Table 1:** Sequences comparative analysis: CA17.67591 versus CA17-G12, Sabin 2, MAD04 and MAD29 strains.

		Genomic region							
		5′UTR	P1^a^	P2^b^	2A	2B	2C	P3^c^	3′UTR
**CA17-G12**	Amino acid (%)	n.a.^d^	**94**	95	91	90	98	98	n.a.
	Nucleotide (%)	88	**78**	80	78	78	81	85	100
**Sabin 2**	Amino acid (%)	n.a.	78	96	95	94	97	97	n.a.
	Nucleotide (%)	81	70	80	80	81	80	84	96
**MAD04**	Amino acid (%)	n.a.	78	98	93	**99**	**100**	99	n.a.
	Nucleotide (%)	81	70	89	79	**93**	**93**	87	99
**MAD29**	Amino acid (%)	n.a.	78	98	95	**97**	**99**	99	n.a.
	Nucleotide (%)	81	70	86	80	**86**	**89**	88	100

a P1: genomic region encoding capsid proteins VP1 to VP4.

b P2: genomic region encoding proteins 2A to 2C.

c P3: genomic region encoding the proteins 3A to 3D^pol^.

d n.a.: not applicable.

Recombination possibility between these three strains was investigated with the bootscanning method [43] (Fig. 2B). These results confirmed that the MAD04 sequences encoding proteins 2B and 2C and the MAD29 2C and 3AB sequences are closely related to those of CA17.67591 and might have been acquired from a recent ancestor of this strain. In contrast, genomic regions encoding viral proteins 3C and 3D^pol^ (P3 region) and the 3′UTR of MAD04 and MAD29 did not show higher similarity with the homologous regions of CA17.67591 than those of the prototype HEV-C strains. These cVDPV sequences may originate from other Madagascar HEV-C strains. From previous results based on partial sequencing of the 3D^pol^ region, the cVDPV MAD04 and certain CA13 strains appeared to have a relatively recent common ancestor [27].

### Molecular cloning of CA17.67591 infectious cDNA and construction of chimeric viruses

**Figure 3:**
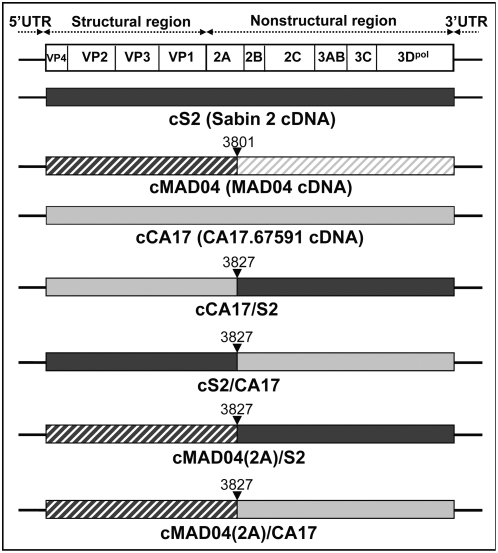
Genomic structure of parental and chimeric viruses generated *in vitro*. The genetic organization of the PV genome is shown (at the top). Closed triangles indicate natural or *in vitro* recombination sites.

To confirm that CA17.67591 could be a recombination partner of PV and to determine main effects of this recombination event on the phenotype of recombinants, we cloned the infectious cDNA of this strain. Although the original isolate differs from the cDNA by a few nucleotides (mentioned in the chapter Materials and Methods), the cDNA-derived virus exhibited phenotypic characteristics similar to those of the original isolate as indicated below. We then used the cloned cDNA together with those of Sabin 2 and MAD04 to construct chimeric viruses (Fig. 3). The recombination site used for these constructs (nt position 3827, according to Sabin 2 nt numbering) was close to the natural site of recombination between mutated Sabin 2 and non-poliovirus sequences (nt 3801) found in MAD04.

The infectivity of the viral RNAs synthesized *in vitro* from the parental and chimeric cDNAs were tested in transfection experiments using HEp-2c cells. Infectivity ranged from 7.5 10^3^ to 4.9 10^4^ pfu/µg of transfected viral RNA, with recombinant cCA17/S2 RNA showing the lowest infectivity rate. These results showed that each RNA synthesized *in vitro* was able to produce viable viruses in HEp-2c cells, and that essential functions of the chimeric cDNAs were not impaired in comparison to their cDNA-derived parental viruses.

### Phenotypic characteristics of chimeric viruses

#### Replication kinetic assays

**Figure 4:**
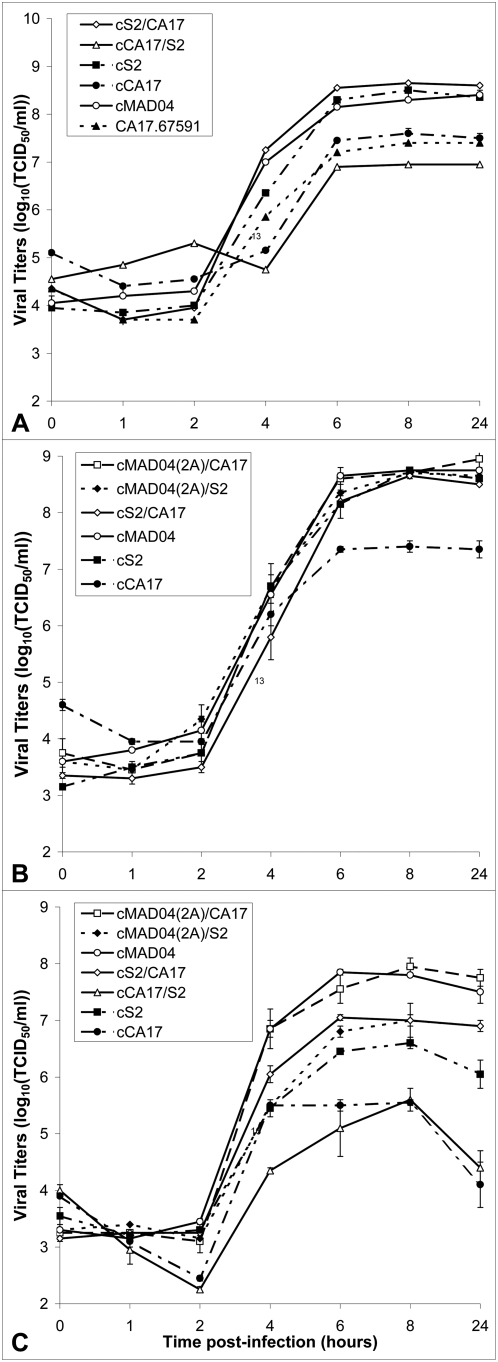
Single-step growth curves of parental and chimeric viruses. HEp-2c cells were infected with the indicated viruses, at a multiplicity of infection of 25 TCID_50_ per cell, and were incubated at 37.0°C (curves A and B) or 40.2°C (curves C). Viruses were harvested at various time points, and titrated. Standard errors of the mean of different samples are represented as error bars.

Replication kinetics of the chimeric cDNA-derived viruses were compared to those of the parental viruses in single-step growth curves (HEp-2c, 37°C). The cDNA-derived CA17 (cCA17) virus replicated as efficiently as the natural CA17.67591 isolate. The final viral yield was slightly lower for the cCA17/S2 virus than for cCA17 (Fig. 4A). However, the growth curves for cS2/CA17, cMAD04(2A)/CA17 and cMAD04(2A)/S2 were similar to those for viruses cS2 and cMAD04 (Fig. 4A and 4B). In HEp-2c cells, viruses bearing the 5′ half of the genomic sequence from CA17.67591 (cCA17 and cCA17/S2) had a lower capacity to replicate than viruses harboring 5′ sequence derived from Sabin 2 (cS2 or cMAD04). The 3′ half of either cS2 or cMAD04 could be replaced by that of cCA17 (in cS2/CA17 and cMAD04(2A)/CA17) without affecting viral replication.

#### Viral plaque assay

**Figure 5:**
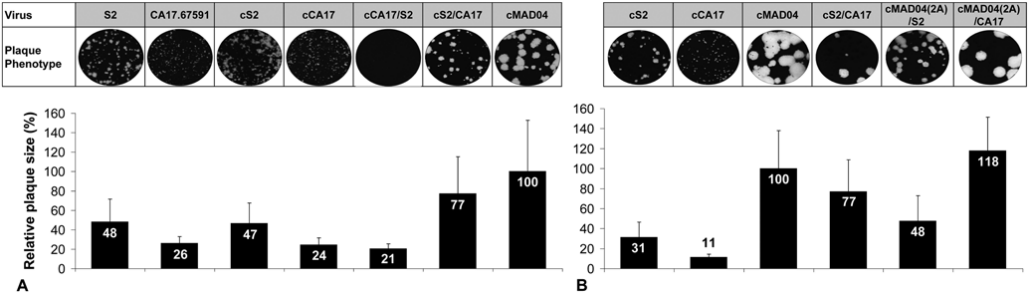
Plaque size of chimeric and parental viruses. Plaque assays were performed on HEp-2c cell monolayers infected with serially diluted viral stocks and incubated at 37°C for 72 h with semi-solid medium. Relative diameters were calculated, taking the average value of cMAD04 plaque diameters to be 100%. Relative plaque sizes (with standard deviations) of two experiments are indicated for viruses in series A and series B. The original Sabin 2 strain (S2) and the corresponding cDNA-derived virus (cS2) were used in series A.

We investigated the capacity of chimeric and parental viruses to spread and form plaques in HEp-2c cell monolayers in a semi-solid medium (Fig. 5). Whereas CA17.65951 isolate and its cDNA-derived counterpart cCA17 formed very small plaques, cMAD04 formed very large plaques (Fig. 5A). The original Sabin 2 strain virus and cS2 formed plaques of an intermediate size. The chimeric cCA17/S2 formed very small plaques similar to those of cCA17, suggesting that the 5′ half of the cCA17 sequence determines the very small plaque size phenotype. Plaques formed by cS2/CA17 and cMAD04(2A)/S2 were of an intermediate size, between those formed by cS2 and cMAD04. cMAD04(2A)/CA17 produced plaques with a similar size to, or even slightly larger than those of cMAD04 (Fig. 5B). Taken together, these findings demonstrated that the 3′ half of the cCA17 genome modulates viral plaque size phenotype. In particular, the recombinant cS2/CA17 formed larger plaques than its parents cS2 and cA17.

#### Temperature sensitivity

As the original PV vaccine strains are temperature sensitive, we compared the replication kinetics of parental and chimeric strains in single-step growth curve experiments in HEp-2c cells at a supra-optimal temperature (40.2°C) (Fig. 4C). The capacity of viruses cCA17 and cCA17/S2 to replicate was lower at this temperature than at 37.0°C. Eight hours post-infection (plateau), titers were 1.3 to 2 log_10_ TCID_50_/ml (tissue-culture infectious dose 50 per ml) lower than at 37.0°C. Significant viral inactivation could be observed between 8 and 24 hours post-infection. Multiplication of cCA17/S2 was slightly slower than that of cCA17. cS2 also had lower titers at 40.2°C than at 37.0°C (plateau values reduced by 2 log_10_ TCID_50_/ml) and showed some inactivation between 8 and 24 hours post-infection. cS2/CA17 replicated more efficiently than cS2 and did not show a drop in titer after 8 hours post-infection. The cS2/CA17 growth curve showed some similarity to that of cMAD04(2A)/S2. The highest final yields were obtained with cMAD04 and cMAD04(2A)/CA17, which were fairly resistant to high temperature, with levels about 0.5 log_10_ TCID_50_/ml lower at 40.2°C than at 37.0°C, 24 hours post-infection.

The temperature sensitivity of viruses was also evaluated by titrating the same viral stock at 37°C and 40.2°C (Table 2). The original Sabin 2 (S2) strain, the CA17.67591 isolate and their cDNA-derived counterparts cS2, cCA17 and the recombinant cCA17/S2 were highly temperature sensitive; indeed, the titer was reduced by ≥3.6 log_10_ TCID_50_/ml at 40.2°C. The differences in titer for cMAD04, cMAD04(2A)/CA17 and the non-temperature sensitive environmental Sabin 2-derived S2 4568 isolate were ≤0.55 log_10_ TCID_50_/ml. cS2/CA17 and cMAD04(2A)/S2 exhibited intermediate differences in titer, of about 1.2 and 1.4 log_10_ TCID_50_/ml, respectively.

**Table 2:** Temperature sensitivity and pathogenicity of parental and chimeric viruses following IC inoculation.

Virus strain	Temperature sensitivity Δlog_10_TCID_50_/ml (37.0°C–40.2°C)^a^	PD_50_ log_10_TCID_50_/mouse^b^
**S2**	3.85±0.05	n.d.^c^
**CA17.67591**	3.6±0.1	>5.6^d^
**cS2**	3.85±0.05	>7.6^d^
**cCA17**	3.85±0.15	>5.6^d^
**cCA17/S2**	4.55±0.05	>5.6^d^
**cS2/CA17**	1.2±0.1	>7.6^d^
**cMAD04**	0.35±0.15	3.8±0.4
**cMAD04(2A)/CA17**	0.45±0.05	3.5±0.4
**cMAD04(2A)/S2**	1.4±0.1	5.0±0.3
**S2 4568**	0.55±0.15	2.7±0.3

a The data are arithmetic means from two experiments; standard errors from the mean are indicated.

b The paralytic doses affecting 50% of mice following inoculation (PD_50_) and standard errors are indicated.

c n.d.: not determined.

d No paralysis occurred following inoculation of the highest doses indicated.

These results showed that the 3′ half of the cCA17 genome renders the chimeric cS2/CA17 less temperature sensitive than the cS2, and makes the cMAD04(2A)/CA17 not temperature sensitive, like MAD04. Potential mutations in the cS2/CA17 viral stock were checked by sequencing at nt positions 481 (5′UTR) and VP1 codon 143. These positions are unstable in vaccinated individuals and are implicated in temperature sensitivity and attenuation [27], [44]–[47]; no unexpected mutations were found.

### Neurovirulence of chimeric viruses

The pathogenicity of chimeric and parental viruses was evaluated in homozygous PVR-Tg21 mice that express the gene encoding the human poliovirus cellular receptor [48],[49]. Following the inoculation of pathogenic polioviruses through parenteral and intranasal (mucosal) routes, these animals developped symptoms similar to those observed in humans (paresis, acute flaccid paralysis or death). In this study, viruses were inoculated intracerebrally (IC) and intranasally (IN).

Viruses with the 5′ half genome from the original CA17 or S2 viruses — i.e. CA17.67591, cCA17, cCA17/S2, cS2, and cS2/CA17 — did not induce paralysis or death in mice at the highest doses inoculated IC (Table 2) or IN. Thus, they were weakly or not pathogenic in adult PVR-Tg21 mice. All other viruses with the 5′ half genome from the VDPVs MAD04, cMAD04, cMAD04(2A)/S2, cMAD04(2A)/CA17, and the highly neurovirulent environmental isolate S2 4568 [50] were pathogenic following IC or IN inoculation.

To determine the dose that induces paralysis or death in 50% of mice (PD_50_), PVR-Tg21 mice were inoculated IC with serial dilutions of viral stocks (Table 2). The PD_50_ of cMAD04 and cMAD04(2A)/CA17 were similar (3.5 and 3.8 log_10_ TCID_50_) but lower than that of cMAD04(2A)/S2 (5.0 log_10_ TCID_50_). S2 4568 was the most pathogenic (PD_50_: 2.7 log_10_ TCID_50_).

**Figure 6:**
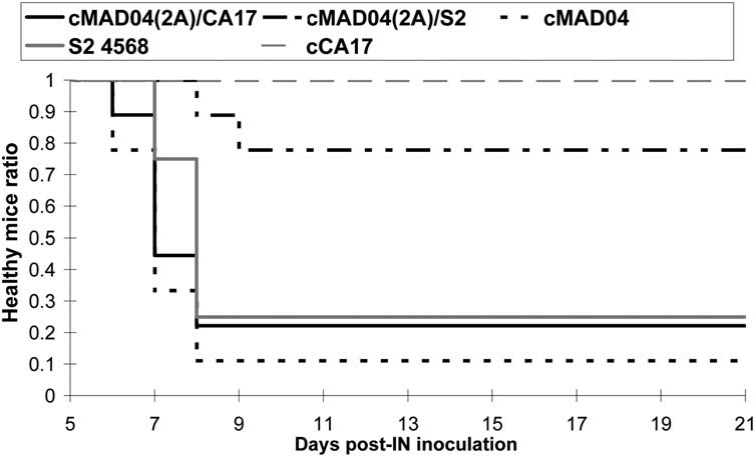
Neurovirulence of viruses inoculated intranasally (IN) in transgenic PVR-Tg mice. A given viral dose (10^6^ TCID_50_ for neurovirulent viruses) was used to inoculate groups of PVR-Tg mice expressing the human poliovirus receptor (9 mice per virus). Animals were checked daily for paralysis and death, for 21 days post-inoculation. Healthy mice ratios following inoculation of parental or chimeric viruses or neurovirulent positive-control virus S2 4568 are shown. No symptoms were observed following the inoculation of the highest doses of cCA17 and cCA17/S2 (10^5^ TCID_50_ per mouse) or cS2 and cS2/CA17 (10^7^ TCID_50_ per mouse) – not shown for the last three viruses.

A given dose of virus was also inoculated IN in PVR-Tg21 mice (Fig. 6). Mice inoculated with cMAD04(2A)/S2 were significantly less affected than those receiving cMAD04, cMAD04(2A)/CA17 and S2 4568 (Log Rank test: *p*<0.02). Pathogenicity of cMAD04(2A)/CA17 did not differ significantly from that of cMAD04 and S2 4568 (*p*>0.4).

These data confirmed that MAD04 neurovirulence is primarily associated with the 5′ half of the genome of mutated Sabin 2 and that the 3′ half of the MAD04 genome modulates the degree of pathogenicity. These results also showed that the 3′ half of the genome from CA17.67591 can replace that from MAD04, rendering the recombinant cMAD04(2A)/CA17 almost as neurovirulent as MAD04.

## Discussion

Most cVDPVs reported so far were recombinants with unidentified non-poliovaccine enteroviruses — either wild PVs or non-PV HEV-C [3], [6]–[13]. Many of these cVDPVs were isolated in countries where wild polioviruses had disappeared, suggesting that these sequences were acquired by genetic recombination with non-PV HEV-C [5]. The co-circulation of such recombinant cVDPVs with genetically related HEV-C Coxsackie A viruses was recently reported in Cambodia and Madagascar [12],[27]. Our studies showed for the first time that non-vaccine sequences present in a recombinant cVDPV can be replaced by homologous sequences of a co-circulating Coxsackie A virus. This provides further evidence that natural recombination occurs between OPV strains and non-PV HEV-C.

The characterization of the *in vitro* engineered MAD04(2A)/CA17 recombinant demonstrated that most of the P2 and P3 regions and the 3′UTR of the cVDPV MAD04 can be exchanged with those of CA17.67591 without significantly affecting phenotypic characteristics, such as viral multiplication in infected cells or pathogenicity in PVR-Tg mice. Additionally, changes in viral phenotype were observed when the 3′ half of the CA17.67591 genome was introduced into the vaccine Sabin 2 cDNA-derived strain cS2. The cS2/CA17 recombinant virus formed larger plaques in semi-solid medium and was much less temperature sensitive than cS2. Primary recombination events can therefore modify the phenotype of recombinants. Provided that the same differences hold true in the human gut, such a Sabin 2/CA17 recombinant could have advantages over Sabin 2 in nature, in terms of multiplication in the infected host, inter-human transmission or both. The viral population may be initially enriched for the newly formed recombinants.

Nevertheless, the cS2/CA17 recombinant was not neurovirulent in PVR-Tg21 mice. This confirmed that the mutated Sabin 2-derived 5′ half of the MAD04 genome, and in particular certain mutations in the 5′UTR ribosomal entry-site (nt 481) and in the VP1 genomic region (codon 143), are essential for pathogenicity [46],[47]. However, the fact that the recombinant MAD04/CA17 was more neurovirulent than MAD04/Sabin 2 suggests that the 3′ half of the CA17.67591 genome acts to promote neurovirulence. Alternatively, the 3′ half of the Sabin 2 genome, could interact with MAD04 to reduce neurovirulence. In any case, recombination between a slightly pathogenic vaccine PV mutant and a HEV-C strain may make the recombinant more pathogenic. In addition, recombination between an original vaccine PV and a HEV-C strain may favor viral multiplication, circulation and therefore genetic drift and acquisition of pathogenic characteristics. Regardless of the sequence of events leading to formation of pathogenic recombinants — whether mutations occur first then subsequent recombination or *vice versa* — recombination between vaccine PV and HEV-C may favor the emergence of cVDPVs.

The contribution of the 3′ half of the CA17 genome to phenotypic differences observed between PV/CA17 recombinants and PV strains remains unclear. Previous studies have shown that the P3-3′UTR region of PV, Coxsackievirus B3 and enterovirus 71 strains is implicated in pathogenicity in animal models and/or in temperature sensitivity of viruses [51]–[54]. It may also modulate diversity among quasispecies by affecting polymerase fidelity, as recently shown for type 1 PV [55]. Recombination between PV and CA17 brings together different genomic fragments. Interactions between these genomic regions may then account for the specific features observed in these recombinants. The intrinsic properties of the 3′ half of CA17 genome and the encoded proteins, and their functional interactions with their PV counterparts, remain to be determined. This will help to elucidate the mechanisms by which recombination modifies the phenotype and gives rise to the emergence of recombinant cVDPVs.

The prototype HEV-C strains CA11, CA13, CA17 and CA20 are the most closely genetically related to PV [14],[15]. Viral isolates belonging to these four serotypes (characterized from VP1 nucleotide sequences) were found in Madagascar in the district of Tolagnaro where the four polio cases caused by MAD04 lineage cVDPVs were identified [11],[27]. The sequencing of the whole genome of one of the CA17 isolates (CA17.67591) which has a high sequence similarity to MAD04 in the 2C region, confirmed its genetic link with the MAD04 lineage and revealed that it is also related to the MAD29 lineage found in another district. We also identified genetic links between this CA17 isolate and the different recombinant cVDPVs lineages isolated in 2005 from other Madagascar districts [10] (Joffret *et al.* unpublished). CA17 strains, or at least viruses related to the CA17.67591 lineage, thus appear to belong to the common recombinant partners of PV in Madagascar. One study showed that the 2BC genomic region of a Sabin3/HEV-C recombinant from Cambodia was related to the 2BC region in indigenous CA17 strains [56]. Given the extensive recombination rate that occurs naturally in all human enterovirus species [4],[14],[20],[25],[26],[28],[57], we cannot exclude the possibility that other HEV-C strains or HEV-C/CA17 recombinants are actually the parents of the Madagascar cVDPVs. However, no such recombinants have been detected among the various HEV-C isolates having been collected in the Tolagnaro district [27]. CA13 isolates from Tolagnaro were also found to be genetically related to MAD04 and MAD29. Recombination of PV with CA13 strains or with CA17/CA13 recombinant strains may be important for certain viral functions and for the emergence of cVDPVs. However, all the phenotypic characteristics of MAD04/CA17 were similar to those of MAD04. This suggests that sequences in the 3D^pol^ region of the cVDPV MAD04, which are related to those of certain co-circulating CA13 strains, do not confer additional selective advantages, at least in terms of the viral functions and characteristics studied.

Whether CA11, CA20 or some other HEV-C are involved in recombination with PV in humans remains unclear. The 2C sequences of a CA20 isolate from Tolagnaro were previously found to be related to those of MAD04 [27]. A recent study of recombinants between the highly pathogenic PV strain Mahoney and a prototype HEV-C (CA20) suggested that the P2 or the P3 regions of the poliovirus genome can be exchanged with those of CA20 without any effect on viral multiplication and, in at least some cases, on pathogenicity in PVR-Tg mice [58]. However, this study demonstrated that recombinants containing the capsid of CA20 (or CA21) and PV sequences are either dysfunctional or non-viable. Combinations of sequences and/or functions derived from PV and other HEV-C may therefore not be compatible and may limit recombinants variety. In this study, the chimera cCA17/S2 (with the 5′UTR and the capsid of CA17.67591) was viable, with multiplication and temperature sensitivity differing only slightly from cCA17. The CA20 - PV Mahoney pair appears to be less permissive for reciprocal genetic exchange than the CA17 – Sabin 2 pair. Unlike a CA20/type 1 PV Mahoney recombinant (with the CA20 capsid), the CA20/type 3 PV Leon counterpart was viable [58], suggesting that some viral factors (phylogenetic distance between viruses and/or functional determinants) may determine the viability and functional properties of recombinants. Nevertheless, small defects in viral function such as those found in CA17/S2 (compared to CA17) may result in selective disadvantages in humans. To the best of our knowledge, CA17/PV recombinants have not yet been isolated from humans.

In conclusion, this study shows that a CA17 isolate co-circulating with a PV/HEV-C recombinant cVDPV can be a recombination partner for PV. Recombination can have a beneficial primary effect on the key phenotypic characteristics of recombinants such as replication and may thus favor the emergence of pathogenic cVDPVs. PVs and CA17 have been recently classified as belonging to the same enterovirus species due to the similarity of their nucleotide sequences; however, these viruses differ greatly in terms of biological properties and pathogenicity in humans. PVs recognize CD155 as its viral receptor [59] and induce a severe paralytic disease. The binding of CA17 to cells seems to be mediated by ICAM1, similarly to the major group of rhinoviruses, agents of the common cold [60]. Moreover, CA17 strains are thought to be poorly pathogenic or not pathogenic at all in humans [61]. The co-circulation of these very different viruses in populations of children [27], and their evolution through intertypic genetic recombination may result in the appearance of more efficiently replicating variants enabling the emergence of new pathogenic lineages. This constitutes an interesting model of viral evolution and emergence.

## Materials and Methods

### Cells and Viruses

HEp-2c cells (derived from a human laryngeal carcinoma cell line) were grown as monolayers in DMEM(1×) high Glucose (with tricine, biotin) (PAA) supplemented with 1% of L-Glutamine 200 mM and 10% new born calf serum.

For the multiplication of viruses containing CA17-derived sequences HEp-2c cell monolayers were grown in DMEM medium containing tryptose phosphate broth and tricine (DMEM-TPB), supplemented with 1% of L-Glutamine 200 mM, 2% of sodium bicarbonate 7.5% and 3% fetal calf serum (FCS).

The coxsackie A virus CA17.67591 strain was isolated in 2002 and grown in HEp-2c cells from stools of healthy child living in the district of Tolagnaro, Madagascar [27].

The cVDPV strain MAD04 was isolated from stool specimens of a patient with poliomyelitis during the 2002 outbreak in Tolagnaro district [11],[27]. The corresponding cDNA-derived virus cMAD04 used in this study was previously described [31].

The poliovaccine virus Sabin 2 (S2) was obtained from the WHO [Behringwerke (S0+1)] “master seeds”. A second passage at 36°C in HEp-2c cells of the original seed was used to prepare viral stocks. The corresponding cDNA-derived cS2 was also used [31].

The PV strain S2/4568 is a non-temperature sensitive and highly neurovirulent Sabin 2 -derived strain isolated from sewage in Israel [50].

The CA17 prototype strain G12 (CA17-G12) was kindly supplied by the National Institute of Public Health and the Environment (RIVM), Bilthoven, The Netherlands and amplified on HEp-2c cells.

### RT-PCR and sequencing

Oligonucleotides used for RT-PCR are presented in Table 3; most polioviruses- and enterovirus-specific primers were previously described [22],[33],[62],[63]. Viral RNA was extracted with QIAamp® Viral RNA Mini Kit (QIAGEN), according to the manufacturer's instructions. Reverse transcription was realised as described by Bessaud et al. [64], using the P1c primer. PCR was carried out in a final volume of 50 µl including 5 µl of Taq Buffer 10× with MgCl_2_, 200 µM of each dNTPs, 10 pmoles of each primers, 5 µl of cDNA and 2.5 U of Taq DNA polymerase (Taq CORE kit 10, Q-Biogen). The thermocycler profile was that of 20 sec at 94°C, followed by 40 cycles of 30 sec at 94°C, 30 sec at 45°C, 1 min at 72°C, and a final elongation step of 10 min at 72°C. PCR products were analysed on ethidium bromide stained agarose gels and purified with QIAquick PCR Purification kit (QIAGEN). The sequences of the resulting amplicons were determined using the BigDye terminator v3.1 kit (Applied Biosystems) and an ABI Prism 3140 automated sequencer (Applied Biosystems) using in most cases primers used for the PCR reaction.

**Table 3:** Oligonucleotides used for RT and PCR.

Genomic region	Name^a^	Sequences 5′->3′ b
		**Poliovirus-specific primers**
5′UTR	UG52	(162) CAAGCACTTCTGTTTCCCCGG (182)
	UC52	(182) CCGGGGAAACAGAAGTGCTTG (162)
	UG53	(578) TGGCTGCTTATGGTGACAAT (597)
	UC53	(596) TTGTCACCATAAGCAGCCA (578)
VP2	UG21	(1177) TCGAGAGGGTGGTGGTGGAA (1196)
	UC21	(1205) TCAGGTAATTTCCACCACCA (1186)
VP3	UC20	(2423) TCATTACACGCTGACACAAA (2404)
	UG1	(2404) TTTGTGTCAGCGTGTAATGA (2423)
2A	UC11	(3503) AAGAGGTCTCTATTCCACAT (3484)
	UG13	(3616) CCCACCTTCCAGTACATGGA (3635)
2C	UG23	(4168) AAGGGATTGGAGTGGGTGTC (4187)
	UC22	(4151) TCAGTAAATTTCTTCAAC CA (4132)
	UG15	(4935) CTGTCACCAACCAGCAAACTT (4955)
	UC15	(4964) CATCTCTTGAAGTTTGCTGG (4945)
3C	UG16	(5920) GTTGGTGGGAACGGTTCACA (5939)
	UC16	(5939) TGTGAACCGTTCCCACCAAC (5920)
3D	UC8	(6375) GATGTCTCTCTTCTTCTTTCCC (6354)
	UG7	(6085) TTTGAAGGGGTGAAGGAACCAGC (6107)
	UG31	(6913) CTGAAAACCTACAAGGGCATAG (6934)
		**Enterovirus-specific primers**
VP1	EUG3a+b	(3004) TGGCAAACT/ATCC/TW/TCC/MAAC/TCC (3023)
2C	EUC2	(4453) TTTGCACTTGAACTGTATGTA (4473)
	EUG19	(4747) AAGGGCATTTTGTTCACGTC (4766)
3A	EUC18	(5237) ATCCATCCTTTCTTCTCACA (5218)
3D	EUG12a+b	(6952) ATGATTGCCTATGGC/GGAT/YGAT/CGT (6974)
	EUC12a	(6974) ACATCGTCMCCATATGCRATCA (6952)
3′UTR	P1c	AGCTGATCGATGGGCTACCATGCGTACCC(T)21C
		**CA17-specific primers**
VP2	CAG1051	(1051) GTTGCCTATGGTCGCTGGCC (1070)
	CAC1422	(1422) CACACCCCCACTTTCACCTGG (1402)
VP3	CAG2285	(2285) GCAACACAGCATACCGGCG (2303)
VP1	CAC2959	(2959) CTTGGTTTCTTGCATGCCCG (2940)
2A	CAG3499	(3499) GTCATGTGGAATAGAGACC (3517)
2B	CAC3891	(3891) CCCAAATGCAGCTCCAAGGG (3872)
3C	CAG5721	(5721) CATCCCCACTCAAATCACCG (5740)
3D	CAC6142	(6142) CGTTCCTGGTCAACACAGCC (6123)
	CAG6837	(6837) GACATACTGTGTCAAGGGCG (6856)
	CAC7040	(7040) GATTGGGCTAGGAGACTAGC (7021)
		**Other**
2C	Mc4171	(4171) CCTTAGCAGCATTACATGC (4153)
5′UTR	OdT	GACCACGCGTATCGATGTCGAC(T)16A

a Most polioviruses- and enterovirus-specific primers have been previously described (see Materials and Methods for references).

b Numbers in brackets refer to the nucleotide positions of Sabin 2 genomic sequences.

except for CA17 specific primers whose numbers refer to CA17.67591 genomic sequences.

The 5′-end of the viral genome was amplified with the 5′/3′ RACEkit (Roche), as described in the manufacturer's protocol. Briefly, viral RNA was used for first-strand cDNA synthesis using the primer UC52; the cDNA was purified and a dA-tailing reaction was carried out. The dA-tailed cDNA was then amplified by PCR using the UC52 and oligo-dT primers.

### Alignment of sequences and genetic analysis

Sequences were aligned and compared using CLC Combined Workbench 3.0 software (CLC bio, Aarhus, Denmark).

Phylogenetic relationships between sequences were inferred by the maximum likelihood method with PUZZLE 4.0, which uses QUARTET PUZZLING as the tree search algorithm [65]. The Hasegawa, Kishino and Yano (HKY) model of substitution for nt with a Ts/Tv of 8.0 was used [66]. Trees were constructed using neighbor-joining of PHYLIP (Phylogeny Inference Package) version 3.6 [67] and branch length given by PUZZLE. The reliability of tree topology was estimated using 10,000 puzzle steps. Trees were drawn with NJ Plot [68].

Nucleotide sequences used for comparative and phylogenetic analysis were PV sequences of PV1-Mahoney, PV2-Lansing, PV3-Leon, Sabin1, Sabin2, Sabin3, MAD04, MAD07, MAD29 (GenBank accession numbers: V01149, M12197, K01392, AY184219, AY184220, AY184221, AM084223, AM084224, AM084225, respectively), nt sequences of prototype strains CA1-Tompkins, CA11-Belgium1, CA13-Flores, CA11v-G9, CA17-G12, CA13v-G13, CA19-NIH8663, CA20-IH35, CA21-Kuykendall, CA21(V)-Coe, CA22-Chulman, CA24v-EH24/70 (Genbank numbers: AF499635, AF499636, AF499637, AF499638, AF499639, AF499640, AF499641, AF499642, AF546702, D00538, AF499643, and D90457, respectively) and, finally, nt sequence of the prototype strain EV70-J670_71 belonging to the human enterovirus of species D (GenBank number: D00820), which was used as outgroup sequence. Former CA15 and CA18 are considered as antigenic variants of CA11 and CA13 [16], and are now named CA11v and CA13v, respectively

Similarity scanning (similarity plots) and bootscanning analysis [43], which depicted relationships among the aligned sequences, were generated using the Simplot software version 3.5.1 [42]. Similarity and bootscanning analysis were performed with a sliding window of 400 nt and a step of 20 nt using Kimura 2 parameters and a transition/transversion ratio of 10. Bootscanning was performed using the neighbour-joining method. Bootscanning consists in the alignment of a possible recombinant sequence with putative parental reference sequences. Bootstrapped phylogenetic trees are built for each segment and finally the bootstrap value for placing the suspected recombinant sequence with the group of a putative parental sequence (CA17.67591 in this study) is plotted along the genome.

Prediction of viral RNA secondary structure was performed using a modified version of MFOLD available in CLC Combined Workbench software [69].

### Construction of parental and chimeric cDNA-derived viruses

The molecular cloning of the Sabin 2 cDNA and the MAD04 cDNA in a modified pBR322 vector (pBR-S2 and pBR-MAD04, respectively) has been previously described [31].

Viral RNA isolated from the CA17.67591 viral stock was reverse transcribed and the cDNA was amplified using PCR as described above except that the Pfu Ultra II Fusion HS DNA polymerase (STRATAGENE) was used.

Two chimeric Sabin 2/CA17.67591 genomes (cS2/CA17 and cCA17/S2) were constructed. Plasmids, oligonucleotides and restriction sites used are given in Table 4. To get the cS2/CA17 cDNA (recombination site at nt 3827), a PCR fragment corresponding to the 3′ half of the CA17 cDNA was amplified with specific primers containing BseRI and NotI cloning restriction sites (Fig. 3) and used to replace the homologous fragment in pBR-S2. To construct cCA17/S2 cDNA, an AscI-BseRI restriction fragment amplified from CA17.67591 by RT-PCR was used to replace the homologous 5′ half of the Sabin 2 cDNA present in pBR-S2. The parental complete CA17 cDNA was reconstituted by replacing the Sabin 2 part of cCA17/S2 cDNA, by the 3′ half of the CA17 cDNA (BseRI-NotI fragment) present in the cloned cS2/CA17 cDNA. This CA17 fragment was also used to replace the Sabin 2 part of the previously described MAD04(2A)/S2 cDNA [31] to give the MAD04(2A)/CA17 cDNA.

**Table 4:** Initial plasmids, oligonucleotides, and restriction sites used for constructing viral infectious cDNAs.

Plasmidic Infectious cDNA	Initial plasmids^a^	Oligonucleotides used for amplifying CA17.67591 sequences^b^		Cloning sites
**pBR-S2/CA17 (11123 bp)**	*pBR-S2 (11123 bp)*	Sense	(3825)5′GGAGCAAGGCATTTCCAATTACATCGAG3′(3852)	BseRI (3827) NotI (7457)
		Antisense	(7473)5′AAGGAAAAAAGCGGCCGCTTTTTTTTTTTTTTTTCTCCGAATTAAAGAAAAATTTA-3′(7418)	
**pBR-CA17/S2 (11141 bp)**	*pBR-S2 (11123 bp)*	Sense	(11092)5′TCGCCGGCGCGCCTAATACGACTCACTATAGGTTAAAACAGCTCTGGGGTTG3′(20)	AscI (11098) BseRI (3827)
		Antisense	(3842)5′TTGGAAATGCCCTGCTCCATAGCTTCCTCCTCATAAGCATACAGGTCTCTAATGTC3′(3787)	
**pBR-CA17 (11141 bp)**	*pBR-CA17/S2 (11141 bp)*	Sense	(3843)5′GGAGCAAGGCATTTCCAATTACATCGAG3′(3870)	BseRI (3844) NotI (7475)
		Antisense	(7491)5′AAGGAAAAAAGCGGCCGCTTTTTTTTTTTTTTTTCTCCGAATTAAAGAAAAATTTA-3′(7436)	
**pBR-MAD04(2A)/CA17 (11141 bp)**	*pBR-MAD04(2A)/S2 (11123 bp)*	Sense	(3825)5′GGAGCAAGGCATTTCCAATTACATCGAG3′(3852)	BseRI (3827) NotI (7457)
		Antisense	(7473)5′AAGGAAAAAAGCGGCCGCTTTTTTTTTTTTTTTTCTCCGAATTAAAGAAAAATTTA-3′(7418)	

a All initial plasmids except pBR-CA17/S2 have been previously described by Riquet et al. [51].

b Numbering refers to the nucleotide sequences of the viral cDNA in the initial plasmid. The nucleotide sequence of the restriction sites used for constructing plasmid are underlined.

All constructs were verified by sequencing and CA17 cDNA sequences were compared to the original CA17.67591 viral stock. The original isolate differs from the cDNA by a few nucleotides (nucleotides 68 G->T, 89 A->T, 2053 U->C, 2401 U->C, 2730 C->T, 2794 G+A->A: Thr+Ala->Thr, 2997 G->A, 3819 U->G, 3825 A->T, 3834 A->G, 3840 C->T, 4302 A->G, 4809 C->T, 6906 C->T, 7339 C->T, respectively).

The T7 RNA^pol^ promoter localised upstream the cloned viral cDNA was used to transcribe infectious RNAs from linearized plasmid (T7 RiboMAX™, PROMEGA). DNA matrixes were eliminated by treatment with RQ1 RNase-Free DNase and viral RNA was purified by phenol/chloroform extraction or with the RNeasy mini kit (QIAGEN). HEp-2c cell monolayers were transfected with purified RNAs (Lipofectamine 2000, INVITROGEN) and incubated at 37°C, 5% CO_2_. Viral stocks were obtained following transfection of 9 µg of RNA/10^6^ cells in DMEM-TPB supplemented with 3% FCS. After almost complete cytopathic effect, viruses were harvested and viral stocks were constituted following two subsequent passages in HEp-2c cells.

### RNA Infectivity assay

The infectivity of the cDNA-derived viral RNAs was tested by transfecting different amounts of viral RNAs in HEp-2c cell monolayers that were subsequently maintained under a 1.2% Avicel (FMC Biopolymer) overlay [70]. Six-well plastic plates were seeded with 2×10^6^ HEp-2c cells per well in DMEM-TPB supplemented with 1% of L-Glutamine 200 mM, 2% of sodium bicarbonate 7.5% and 3% FCS and kept in an incubator at 37°C and 5% CO_2_ for about 24 h. Cell monolayers were washed twice with DMEM without serum, transfected using Lipofectamine 2000 with 1 µg of decimally diluted RNA per well and incubated at 37°C and 5% CO_2_ for 30 min. Then, the inoculum was removed and replaced by 2 ml per well of DMEM-TPB supplemented with 1% of L-Glutamine 200 mM, 2% of sodium bicarbonate 7.5% without serum before incubation at 37°C and 5% CO_2_ for 1 h. Next, the medium was removed and replaced by 3 ml of a mixture containing 1 volume of 2.4% Avicel in water and 1 volume of DMEM 2× supplemented with 2% L-glutamine 200 mM, 2% penicillin/streptomycin 100×, 4% tricine 1 M pH 7.4, 3% sodium bicarbonate 7.5%, 5% MgCl_2_ 1 M and 4% FCS. Plaques were incubated at 37°C in a 5% CO_2_ incubator for 3 days. Avicel-containing medium was then removed; cells were washed twice with PBS (without CaCl_2_, MgCl_2_) and stained. The number of plaque forming unit (pfu) per µg of transfected RNA was calculated (mean of two experiments).

### Viral plaque assay

Six-well plastic plates seeded with HEp-2c cells were prepared as described in the RNA infectivity assay. They were infected with 500 µl per well of decimally diluted viral stocks. Following washing, incubation under Avicel at 37°C for 3 days and staining, the diameter of all isolated plaques was measured and a mean plaque diameter and standard deviation (two experiments) were calculated.

### Replication kinetic assay

Replication kinetics of parental and chimeric viruses were compared in single-step growth experiments. HEp-2c cells were infected at a multiplicity of infection of 25 TCID_50_ per cell. After adsorption for 30 min, cells were washed twice and incubated at 37.0°C in a 5% CO2 incubator or at 40.2°C in a water bath. Then, infected cells were frozen at various time points post-infection. Viral titers were determined for each time (TCID_50_/ml).

### Temperature sensitivity

In addition to single-step growth at 40.2°C, the temperature sensitivity of viruses was also evaluated by titrating the same viral stock (TCID_50_/ml) at various temperatures in DMEM-TPB containing 2.2% FCS. One plate was incubated at 37.0°C (optimal temperature) in a 5% CO_2_ incubator and the second was incubated at 40.2°C (supra-optimal temperature) in a water bath. After 5 days of incubation, cells were stained and the titer (TCID_50_/ml) was calculated for each temperature. Results are expressed as the difference between the viral titre measured at 37.0°C and that at 40.2°C.

### Assay of neurovirulence in PVR-Tg mice

Virus neurovirulence was tested in homozygous PVR-Tg21 mice that constitutively express the human poliovirus receptor CD155 (generous gift from A. Nomoto) [48]. To determine PD_50_ groups of 6 six-week-old PVR-Tg21 mice (equal number of males and females) were inoculated IC with a given amount of each virus ranging from 10^3^ to 10^7^ TCID_50_/ml in DMEM containing 0.1% fetal calf serum (30 µl per mouse). Mice were examined daily for 21 days post-inoculation for paralysis and/or death and the PD_50_ was calculated by the method of Reed and Muench [71]. Standard errors were determined according to the formula of Pizzi [72].

To evaluate neurovirulence a given dose of virus was also inoculated IN (2×5 µl per mouse) in groups of PVR-Tg21 mice (5 males and 4 females or 3 males and 3 females). Survival curves were determined according to the Kaplan-Meir method and compared using the Log Rank test with the XLSTAT software version 2008.6.05 (ADDINSOFT).

Viruses S2 4568 and cS2 were used as neurovirulent and non-pathogenic controls, respectively. To confirm the inoculated dose, viral inocula were back-titrated following inoculation.

All experiments were conducted in full compliance with French regulations regarding laboratory animal welfare. Protocols were approved by the Veterinary Staff of the Central Animal Facility of Institut Pasteur. Before IC and IN inoculation mice were anesthetized by intraperitoneal injection of 100 µl of PBS containing 0.25 µg of Xylazine (Rompun, BAYER) and 2.5 µg Ketamine (Imalgene, MERIAL).

### Virus blocking assay

To test whether infection of HEp-2c cells by CA17.67591 was mediated by ICAM-1, we used a monoclonal antibody (mAb) against this molecule (clone 8.4 A6, Sigma) [40]. A mAb against the PV receptor CD155 (mAb. 404, kind gift of Marc Lopez, Marseille) was used as control [41]. Briefly, 2-days old confluent HEp-2c cells in 96-well plates were treated either by anti-ICAM-1, or anti-CD155 mAbs, or mock-treated for 2 h at 37° before infection, as described by Minor and coll. [73]. Each well containing about 40,000 cells was then infected by 10^3^ TCID_50_ of either CA17.67591, or Sabin 2 cDNA-derived strains, or mock-infected, in the presence of antibodies as described [73]. Cell protection was evaluated under the microscope 4 days post-inoculation.
